# Investigating Pre-pregnancy and Late-Pregnancy E-cigarette Use Exposure and Gestational Diabetes Mellitus

**DOI:** 10.7759/cureus.95936

**Published:** 2025-11-02

**Authors:** Elizabeth R Burgess, Hope M Cherian, Carter Wegner, Grettel Castro, Marcia Varella

**Affiliations:** 1 Obstetrics and Gynecology, Florida International University, Herbert Wertheim College of Medicine, Miami, USA; 2 Humanities, Health, and Society, Florida International University, Herbert Wertheim College of Medicine, Miami, USA; 3 Translational Medicine, Florida International University, Herbert Wertheim College of Medicine, Miami, USA; 4 Medical and Population Health Sciences Research, Florida International University, Herbert Wertheim College of Medicine, Miami, USA

**Keywords:** e-cigarette use, gestational diabetes mellitus (gdm), maternal risk factors, pregnancy outcomes, pregnancy risk assessment monitoring system (prams)

## Abstract

Background and objective

E-cigarette (EC) use is on the rise, particularly among young adults, yet its impact on pregnancy remains poorly understood. Among women of reproductive age, rising EC use has raised concerns about adverse outcomes in pregnancy, including gestational diabetes mellitus (GDM). More than 40% of pregnant smokers report using non-cigarette nicotine products, but clinical guidance for EC use during pregnancy is limited. While prior studies suggest associations between EC use and adverse fetal outcomes, maternal risks have been sparsely explored. This study aimed to investigate whether EC use pre- or during pregnancy is associated with GDM in U.S. women with singleton births.

Methods

This was a secondary analysis of Pregnancy Risk Assessment and Monitoring System data (2016-2021). All data were based on self-reported responses collected from women after the birth of their index child. Self-reported EC use was categorized as follows: (1) three months pre-pregnancy, (2) last three months of pregnancy, (3) both periods, or (4) no use. GDM diagnosis (ever/never) was also self-reported. Logistic regression models were employed to estimate odds ratios (ORs) and 95% confidence intervals (CIs), adjusting for demographic, socioeconomic, and pregnancy-related factors, using Stata 17.

Results

Among 204,757 women with singleton births, <5% reported EC use, with 72% quitting before the third trimester. Overall, 7.7% developed GDM. In unadjusted models, EC use pre-pregnancy was not associated with GDM (OR: 1.0; 95% CI: 0.86-1.16), while EC use during both pre-pregnancy and in the third trimester was linked to lower odds (OR: 0.71; 95% CI: 0.54-0.93). Adjusted models showed that EC use pre-pregnancy was associated with increased GDM risk (adjusted OR: 1.27; 95% CI: 1.07-1.51; p = 0.006), with no significant associations observed for other categories.

Conclusions

EC use pre-pregnancy was linked to higher GDM odds, after adjustment for potential confounders. These findings underscore the need for further research on timing, frequency, and health effects to inform targeted public health interventions. Understanding the effects of EC use during pregnancy is crucial for the well-being of pregnant women and their unborn children, as well as to prevent adverse pregnancy outcomes. While there have been some analyses of associations between EC use during pregnancy and fetal birth complications, there is little information about its association with maternal complications during pregnancy and labor. This research adds to the body of evidence-based information that can inform policy decisions and guideline development. More importantly, it provides preliminary insights that will help healthcare professionals more effectively counsel patients and evaluate associated risks.

## Introduction

The use of e-cigarettes (EC) has surged in popularity across the United States, particularly among adolescents and young adults. While an increasing body of evidence highlights the harmful effects of EC use in these populations, its impact on pregnant women remains a relatively understudied area of research [1-3]. Recent data estimate that over 40% of pregnant women who smoke conventional tobacco products also use non-cigarette or nicotine delivery products [4]. While smoking cessation during pregnancy is highly encouraged, limited guidance exists regarding EC use specifically, leaving many pregnant women without clear recommendations.

The risks associated with traditional cigarette smoking during pregnancy are well-documented and include an increased likelihood of adverse fetal outcomes, such as preterm birth, low birth weight, intrauterine growth restriction (IUGR), and intrauterine fetal death [5]. Maternal risks, including ectopic pregnancy, placental abruption, placenta previa, preterm premature rupture of membranes, and gestational diabetes mellitus (GDM), have also been described [5,6]. Notably, GDM carries significant health implications for both the pregnant woman and the child, including an elevated long-term risk of type 2 diabetes [7]. Given that nicotine and other chemicals in ECs have been implicated in metabolic changes, such as increased insulin resistance, they may plausibly contribute to GDM risk [8].

Previous research utilizing the Pregnancy Risk Assessment Monitoring System (PRAMS) database has primarily examined fetal outcomes associated with EC use during pregnancy, including low birth weight, preterm birth, and small-for-gestational-age (SGA) infants [9-13]. Studies report increased risks of adverse neonatal outcomes among EC users, with adjusted risk ratios for low birth weight and preterm birth ranging from 1.38 to 1.88, and similar findings for SGA outcomes [9-12]. However, no significant association has been observed between EC use during pregnancy and low gestational weight gain (adjusted odds ratio (OR): 0.99, 95% confidence interval (CI): 0.78-1.27) [13]. Despite these insights, research on maternal outcomes, particularly GDM, remains scarce.

To address this gap, our study aims to assess whether EC use pre- and/or during pregnancy is associated with GDM. By investigating this relationship, we seek to contribute to a more comprehensive understanding of the maternal health effects of EC use during pregnancy.

## Materials and methods

Study design and population

This study involved a secondary analysis of data from a cross-sectional study database, the PRAMS. The study population included U.S. women who had singleton births and participated in the PRAMS database from 2016 to 2021 (Phase 8). All data were based on self-reported responses collected from women after the birth of their index child. Participants with missing data on key variables, including outcomes and exposure, or those reporting answers as unknown, were excluded. PRAMS data is de-identified; thus, it is considered non-human subject research and does not require Institutional Review Board (IRB) approval.

The primary exposure variable was EC use, collected by PRAMS questionnaires if occurring during two distinct time periods: either three months pre-pregnancy or in the last three months of pregnancy. Based on these variables, participants were categorized into four EC exposure groups: (1) non-user, defined as no EC use in either period; (2) last trimester only, defined as no use pre-pregnancy but any use during the last three months of pregnancy; (3) pre-pregnancy only, defined as any use before pregnancy but no use during the last trimester; and (4) pre-pregnancy and last trimester, defined as any use in both periods. The primary outcome variable was being diagnosed with GDM during this pregnancy.

Covariates and potential confounders included maternal sociodemographic characteristics (age, race/ethnicity, education, marital status, and household income), pregnancy-related factors (pregnancy intention, Kotelchuck index of prenatal care adequacy, prenatal vitamin use, parity, and history of preterm birth), maternal health behaviors (pre-pregnancy BMI, multivitamin use frequency, alcohol consumption, and cigarette use during pregnancy), and delivery characteristics (delivery method and year of delivery). Participation in the Special Supplemental Nutrition Program for Women, Infants, and Children (WIC) was also considered. These factors were adjusted for in the analysis to account for their potential confounding effects.

Statistical analysis

Statistical analyses were conducted to address the study objectives using Stata 17, accounting for the complex survey design. Univariate analyses were performed to describe the sample characteristics and assess patterns of missing data. Bivariate analyses were conducted, and chi-square tests were used to assess statistical differences in participant characteristics across exposure status. Additional bivariate analyses were performed to evaluate the association between covariates and the frequency of complications. Variables with p-values less than 0.2 for differences according to exposure and outcome were considered potential confounders and included in models for adjustment. Following an assessment of collinearity, multiple logistic regression analyses were performed to estimate crude and multivariable-adjusted ORs and 95% CIs for the association of interest.

## Results

**Figure 1:**
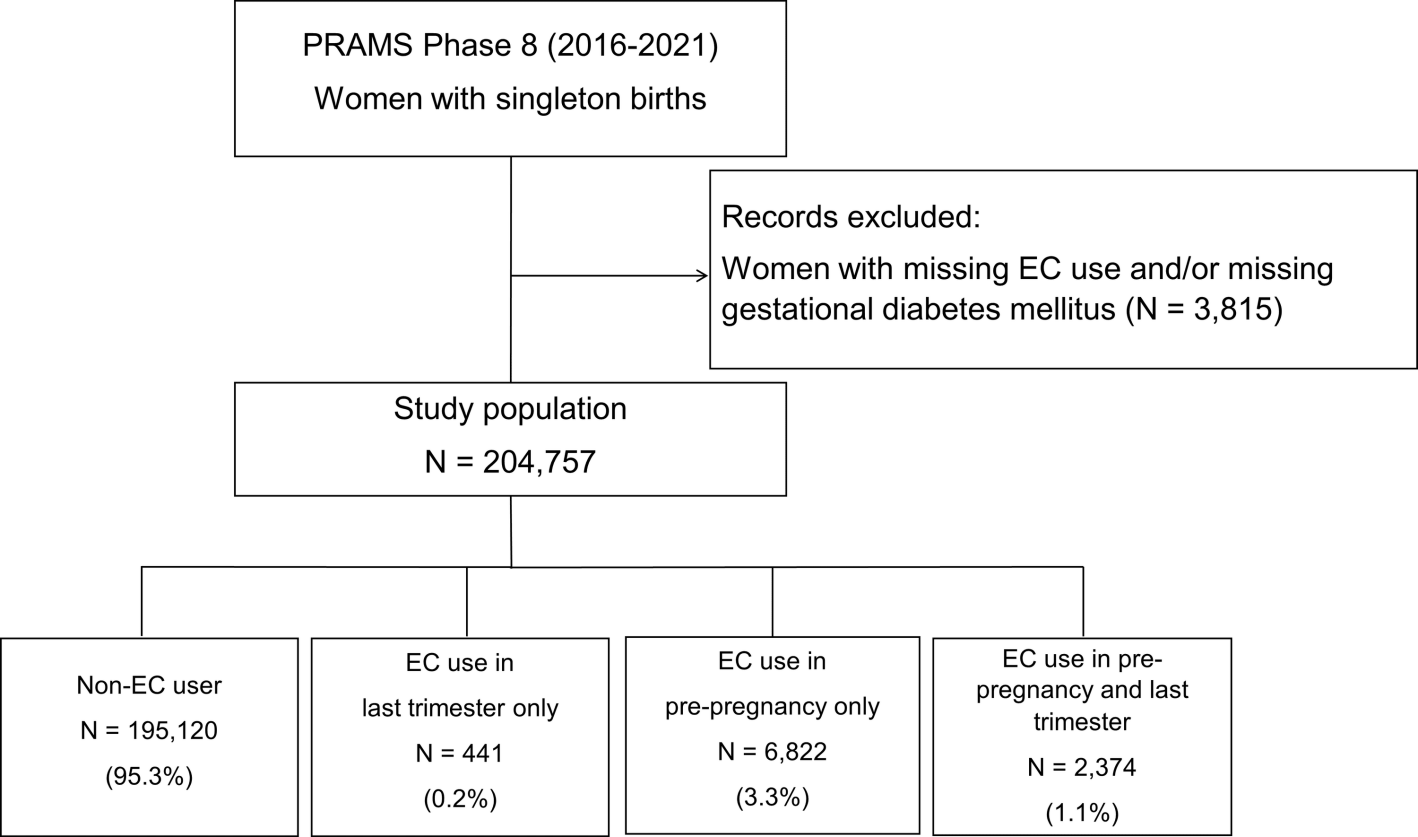
Flow diagram of the the primary analytic dataset and the planned subgroup analysis Numbers presented are unweighted sample sizes PRAMS: Pregnancy Risk Assessment Monitoring System; EC: e-cigarette

A flowchart of the selected study population is depicted in Figure 1. A total of 221,381 women were included in the PRAMS database Phase 8 (2016-2021). Of them, 12,809 women did not meet the inclusion criteria for this study due to multiple births. Of the remainder, 3,815 had missing data for the main exposure or outcome variables. Therefore, our study sample consisted of 204,757 women. Overall, 95.3% women reported no EC use (n = 195,120), 0.2% reported EC use in the last trimester only (n = 441), 3.3% reported EC use pre-pregnancy only (n = 6,822), and 1.1% reported EC use pre-pregnancy and in the last trimester (n = 2,374) (Figure 1).

Maternal sociodemographic and health behavior characteristics of the sample are presented in Table 1. In all EC use categories, most participants identified as White, non-Hispanic women and demonstrated an adequate or adequate plus Kotelchuck index. Additionally, most women delivered their first or second child, had no history of preterm delivery, had BMIs categorized as normal or overweight, and delivered vaginally. Compared to non-users, EC users either pre- and/or during pregnancy were more often younger (18-24 years), had lower educational attainment (high school level), and were unmarried. Those who reported EC use pre- and/or during pregnancy also had a higher proportion of unintentional pregnancies, were less likely to take prenatal vitamins, and reported a lower income. Lastly, compared to non-EC users, women who reported EC use during the last trimester of pregnancy were more likely to be WIC participants and use traditional cigarettes, and women who reported EC use only pre-pregnancy also consumed a higher number of alcoholic drinks per week pre-pregnancy.

**Table 1:** Characteristics of women with live singleton births from 2016–2021 who participated in PRAMS, stratified by e-cigarette use behavior ^a^Unweighted. ^b^Weighted Percentages are calculated based on available data for each variable (missing responses excluded) PRAMS: Pregnancy Risk Assessment Monitoring System; BMI: body mass index

Characteristics among PRAMS participants with live singleton births									
	Electronic cigarette use status and use timing								
Characteristics	Non-user (n = 195,120)		Last trimester only (n = 441)		Pre-pregnancy only (n = 6,822)		Pre-pregnancy and last trimester (n = 2,374)		P-value
	No.^a^	%^b^	No.^a^	%^b^	No.^a^	%^b^	No.^a^	%^b^	
Maternal age at delivery (years)									<0.001
≤17	2,195	1.1	0	0	175	2.4	56	2.3	
18-24	39,022	20	136	31.3	2914	42.9	806	34.8	
25-29	54,561	28.9	143	30.1	1801	27.6	683	29.9	
30-34	57,642	30.7	97	23.7	1185	18	510	22.9	
≥35	36,951	19.4	58	14.9	584	9.1	264	10.2	
Pre-pregnancy BMI category									<0.001
Underweight	6,339	3.2	29	3.7	360	4.7	163	6.1	
Normal	81,506	44.8	201	48.4	2837	43	1009	43.5	
Overweight	47,791	25.9	99	25.2	1611	24.3	564	35.6	
Obese	50,370	26	98	22.7	1880	28.1	578	24.8	
Maternal race/ethnicity									<0.001
White	111,399	68.9	340	86.9	4550	81.3	1698	86.8	
Black	36,017	16	35	6.5	689	7.8	202	5.5	
Asian	13,334	5.8	3	0.1	149	2	30	1	
Other	27,893	9.3	55	6.5	1231	8.9	376	6.7	
Hispanic									<0.001
Not Hispanic	156,618	82.4	412	95.7	5845	89.7	2125	92.2	
Hispanic	37,488	17.6	27	4.3	947	10.3	240	7.8	
Maternal education									<0.001
≤8th grade	5,854	3.2	3	0.1	54	0.8	28	0.9	
9-12th grade	17,179	8.1	66	19.3	900	11.9	451	18.8	
High school graduate	45,883	24	179	36.5	2468	38.4	981	42.6	
Some college	54,150	26	161	37.6	2488	34.4	728	30.3	
Bachelor's or higher	70,273	38.7	31	6.5	861	14.6	161	7.4	
Married									<0.001
Married	118,330	63.3	122	25.2	2393	36.3	695	29.7	
Other	76,652	36.7	319	74.8	4418	63.7	1668	70.3	
Income									<0.001
$0 to $20,000	49,376	24.4	234	54.8	2582	37.4	1183	53.6	
$20,001 to $33,000	26,044	14.2	78	15.7	1261	19.6	418	16.6	
$33,001 to $50,000	28,129	15.8	56	18	1053	17.3	314	14.3	
$50,001 to $73,000	16,123	8.9	15	2.2	510	9.2	114	6.1	
$73,001 to $100,000	10,814	5.9	12	1.6	261	4.5	53	2.4	
WIC recipient									<0.001
Yes	69,434	32.9	239	57.8	3094	43.1	1242	51.8	
No	12,2838	67.2	194	42.2	3640	56.9	1089	48.2	
Kotelchuck index									<0.001
Inadequate	23,913	12	87	23	974	14	490	19	
Indeterminant	20,084	10.8	44	9.1	693	10.8	270	11	
Adequate	83,046	46.6	154	38.2	2767	44.1	802	39.3	
Adequate +	62,576	30.6	148	29.8	2208	31.1	747	30.7	
Pregnancy intention									<0.001
Intentional	113,875	61.1	144	34.2	2798	43.7	887	38	
Not intentional	46,867	23.8	163	36.9	2330	33.7	819	36.7	
Unsure	31,251	15.1	123	28.9	1607	22.6	630	25.4	
Traditional smoking									<0.001
Yes	13,339	5.8	255	57.2	1440	19.5	1084	43.1	
No	180,606	94.2	180	42.8	5316	80.5	1248	56.9	
Pre-pregnancy alcohol drinks (number per week)									<0.001
No drinking	88,104	43.3	168	38.9	1563	21.7	897	39.2	
<1	50,447	27.2	109	23.5	1908	28.4	643	28	
01-Jul	50,126	27	120	28.5	2717	41.2	632	26.9	
>7	4,930	2.5	43	9.2	597	8.7	175	5.9	
Prenatal vitamin use (times per week)									<0.001
Didn’t take	97,645	49.2	343	81.7	4644	68.2	1639	72.5	
01-Mar	14,380	7.2	20	5	445	6.2	192	7.7	
04-Jun	12,187	6.3	17	3.7	285	3.9	87	2.4	
Every day	69,882	37.3	59	9.6	1422	21.7	444	17.4	
Parity									<0.001
Primiparous	76,268	38.5	157	32.1	3785	55.8	926	41.4	
2nd child	61,426	33.2	125	32.9	1660	25.2	651	26.6	
3rd child	31,897	16.5	81	15.5	818	12.4	446	19.4	
4-6th child	22,806	10.7	72	18	507	6.3	310	11.9	
7th child +	2,392	1.1	5	1.6	37	0.4	33	0.7	
Preterm birth history									<0.001
Yes	9,371	3.7	25	3.1	264	2.5	169	5.2	
No	185,656	96.3	416	96.9	6557	97.5	2204	94.8	
Delivery method									0.57
Vaginal	133,011	70.1	291	67.6	4707	70.2	1599	68.3	
C-section	61,994	29.9	149	32.4	2112	29.8	773	31.7	
Year of delivery									<0.001
2016	27,070	14.3	63	12.5	809	11.1	286	11	
2017	30,575	16.1	60	10.5	830	13.2	293	13.7	
2018	36,474	18.5	76	19.5	1009	14.5	384	15.7	
2019	37,411	18	80	17.5	1285	17.9	430	15.9	
2020	34,470	17.1	79	20.9	1475	20.9	451	18.8	
2021	29,120	15.9	83	23.2	1414	22.4	530	24.9	

Maternal GDM frequency in the sample is summarized in Table 2. Overall, 92.7% of women did not have GDM (n = 189,020), and 7.31% had GDM (n = 15,737). The prevalence of GDM increased significantly with maternal age, ranging from 1.3% (n = 38) among those aged ≤17 years to 11.7% (n = 4,677) in women aged 35 or older (Table 2). Asian women had the highest prevalence of GDM (15.4%, n = 2,030), followed by women categorized as Other (8.7%, n = 2,655), White (6.7%, n = 7,867), and Black (6.2%, n = 2,336). Hispanic ethnicity was associated with a higher prevalence of GDM (8.6%, n = 3,281) compared to non-Hispanic women (7.0%, n = 12,374). An inverse relationship was observed with education level, with the highest prevalence of GDM seen with education levels ≤8th grade (n = 649). Parity and pre-pregnancy BMI were also significant contributors, with GDM prevalence rising in women with more children and higher BMIs (obese women had 12.3%, n = 6,758, of GDM compared to 4.3%, n = 3,858, in normal-weight women). Lastly, women who delivered via C-section had a higher prevalence of GDM (9.5%, n = 6,364) compared to vaginal delivery (6.4%, n = 9,361) (Table 2).

**Table 2:** Frequency of gestational diabetes according to selected characteristics ^a^Unweighted. ^b^Weighted Percentages are calculated based on available data for each variable (missing responses excluded) BMI: body mass index; WIC: The Special Supplemental Nutrition Program for Women, Infants, and Children

	Gestational diabetes occurrence				
	Yes (n = 15,737)		No (n = 189,020)		P-value
	No.^a^	%^b^	No.^a^	%^b^	
Delivery age (years)					<0.001
≤17	38	1.3	2,388	98.7	
18-24	1,536	3.4	41,342	96.6	
25-29	3,619	6.3	53,569	93.7	
30-34	5,172	8.4	54,262	91.6	
35+	4,677	11.7	33,180	88.3	
Pre-pregnancy BMI category					<0.001
Underweight	240	3.5	6,651	96.6	
Normal	3,858	4.3	81,695	95.7	
Overweight	3,946	7.7	46,119	92.3	
Obese	6,758	12.3	46,168	87.7	
Race					<0.001
White	7,867	6.7	110,120	93.3	
Black	2,336	6.2	34,607	93.9	
Asian	2,030	15.4	11,486	84.6	
Other	2,655	8.7	26,900	91.3	
Hispanic					<0.001
Hispanic	3,281	8.6	35,421	91.4	
Not Hispanic	12,374	7	152,627	93	
Maternal education					<0.001
≤8th grade	649	9.5	5,290	90.5	
9-12th grade	1,349	7.6	172,247	92.4	
High school graduate	3,542	6.8	45,969	93.2	
Some college	4,699	7.9	52,828	92.1	
Bachelor's +	5,378	7	65,948	93	
Married					<0.001
Married	10,297	7.9	111,243	92.1	
Other	5,429	6.4	77,628	93.6	
Income					<0.001
$0 to $20,000	3,530	6.6	49,845	93.4	
$20,001 to $33,000	2,288	7.5	25,513	92.5	
$33,001 to $50,000	2,554	8.2	26,998	91.8	
$50,001 to $73,000	1,487	8.3	15,275	91.7	
$73,001 to $100,000	951	8.2	10,189	91.8	
$100,001 and above	3,588	6.9	44,912	93.1	
WIC recipient					0.007
Yes	5,795	7.6	68,214	92.4	
No	9,700	7.1	118,061	92.9	
Kotelchuck index					<0.001
Inadequate	1,760	6.9	23,704	93.1	
Indeterminant	1,089	4.5	20,002	95.5	
Adequate	5,284	5.8	81,485	94.2	
Adequate +	7,171	10.8	58,508	89.2	
Pregnancy intention					<0.001
Intentional	9,645	7.7	108,059	92.3	
Not intentional	3,291	6.4	46,888	93.6	
Unsure	2,511	7.2	31,100	92.8	
Traditional smoking					0.611
Yes	1,177	7.1	14,941	92.9	
No	14,457	7.3	172,893	92.7	
Pre-pregnancy alcohol drink (number per week)					<0.001
No drinking	7,728	8.1	83,004	91.9	
<1 drink	4,146	7.4	48,961	92.6	
1-7 drinks	3,363	6.1	50,232	93.9	
>7 drinks	374	6.6	5,371	93.4	
Prenatal vitamin use (times per week)					0.005
Didn’t take	7,916	7.3	96,355	92.7	
1-3 times	1,167	7	13,870	93	
4-6 times	873	6.4	11,703	93.6	
Every day	5,685	7.6	66,122	92.4	
Parity					<0.001
Primiparous	5,417	6.4	75,719	93.6	
2nd child	4,711	7.1	59,151	92.9	
3rd child	2,893	8.4	30,349	91.6	
4-6th child	2,421	9.7	21,274	90.3	
7th child +	280	9.8	2,187	90.2	
Preterm birth history					<0.001
Yes	1,028	11.6	8,801	88.4	
No	14,700	7.1	180,133	92.9	
Delivery method					<0.001
Vaginal	9,361	6.4	130,247	93.6	
C-section	6,364	9.5	58,664	90.5	
Year of delivery					<0.001
2016	1,927	6.6	26,301	93.5	
2017	2,283	6.9	29,475	93.1	
2018	2,684	6.7	35,259	93.3	
2019	3,000	7.3	36,206	92.7	
2020	3,145	8.2	33,330	91.9	
2021	2,698	8.1	28,449	91.9	

Table 3 presents the analysis of potential associations between maternal EC use, selected characteristics, and the occurrence of GDM. Before adjusting for potential confounders, there were no associations between those using EC only in the last trimester or only pre-pregnancy and GDM. However, the odds of GDM were 29% lower in women with EC use both pre- and during pregnancy, as compared to non-users (95% CI: 0.54-0.93, p = 0.014). However, after adjusting for multiple confounders, this association was no longer statistically significant for the EC use in both the pre- and during-pregnancy groups (Table 3). After adjustments for confounders, the odds of GDM were 27% higher in those with EC use pre-pregnancy and quit before their third trimester, as compared to non-users (95% CI: 1.07-1.51, p = 0.006). No other statistically significant associations were found between GDM and the remaining EC use categories.

**Table 3:** Associations between maternal e-cigarette use status, selected characteristics, and GDM occurrence GDM: gestational diabetes mellitus; OR: odds ratio; CI: confidence interval; BMI: body mass index; WIC: The Special Supplemental Nutrition Program for Women, Infants, and Children

	Unadjusted		Adjusted	
	OR (95% CI)	P-value	OR (95% CI)	P-value
Electronic cigarettes user categories				
Non-user	Reference	Reference	Reference	Reference
Last trimester only	1.02 (0.58, 1.78)	0.946	1.11 (0.59, 2.11)	0.733
Pre-pregnancy only	1.00 (0.86, 1.16)	0.985	1.27 (1.07, 1.51)	0.006
Pre-pregnancy and last trimester	0.71 (0.54, 0.93)	0.014	0.93 (0.69, 1.25)	0.622
Delivery age, years				
≤17	0.20 (0.12, 0.33)	<0.001	0.18 (0.08, 0.40)	<0.001
18-24	0.53 (0.48, 0.58)	<0.001	0.52 (0.46, 0.58)	<0.001
25-29	Reference	Reference	Reference	Reference
30-34	1.37 (1.28, 1.47)	<0.001	1.44 (1.33, 1.55)	<0.001
≥35	1.99 (1.86, 2.14)	<0.001	1.94 (1.78, 2.12)	<0.001
Pre-pregnancy BMI category				
Underweight	0.80 (0.65, 0.98)	0.033	0.81 (0.64, 1.02)	0.073
Normal	Reference	Reference	Reference	Reference
Overweight	1.86 (1.74, 2.00)	<0.001	1.82 (1.68, 1.97)	<0.001
Obese	3.12 (2.93, 3.33)	<0.001	3.07 (2.84, 3.31)	<0.001
Race				
White	Reference	Reference	Reference	Reference
Black	0.92 (0.85, 0.99)	0.022	0.80 (0.72, 0.88)	<0.001
Asian	2.55 (2.35, 2.76)	<0.001	2.93 (2.66, 3.24)	<0.001
Other	1.34 (1.23, 1.45)	<0.001	1.19 (1.07, 1.31)	<0.001
Hispanic				
Hispanic	1.24 (1.17, 1.32)	<0.001	1.17 (1.07, 1.28)	<0.001
Not Hispanic	Reference	Reference	Reference	Reference
Maternal education				
≤8th grade	1.45 (1.26, 1.66)	<0.001	0.99 (0.80, 1.22)	0.900
9-12th grade	1.13 (1.02, 1.25)	0.023	1.19 (1.04, 1.37)	0.011
High school grad	Reference	Reference	Reference	Reference
Some college	1.18 (1.10, 1.27)	<0.001	1.02 (0.94, 1.12)	0.619
Bachelor's and higher	1.04 (0.97, 1.11)	0.281	0.84 (0.76, 0.93)	<0.001
Married				
Married	Reference	Reference	Reference	Reference
Other	0.80 (0.76, 0.84)	<0.001	0.91 (0.84, 0.99)	0.028
Income				
$0 to $20,000	Reference	Reference	Reference	Reference
$20,001 to $33,000	1.16 (1.06, 1.26)	<0.001	1.02 (0.92, 1.13)	0.656
$33,001 to $50,000	1.27 (1.16, 1.38)	<0.001	1.12 (1.00, 1.25)	0.044
$50,001 to $73,000	1.28 (1.16, 1.42)	<0.001	1.11 (0.97, 1.27)	0.114
$73,001 to $100,000	1.26 (1.12, 1.42)	<0.001	1.15 (0.98, 1.33)	0.079
$100,001 and above	1.05 (0.98, 1.14)	0.185	0.99 (0.87, 1.12)	0.832
WIC recipient				
Yes	1.08 (1.02, 1.14)	0.007	1.12 (1.03, 1.21)	0.007
No	Reference	Reference	Reference	Reference
Kotelchuck index				
Inadequate	1.21 (1.11, 1.33)	<0.001	1.24 (1.11, 1.38)	<0.001
Indeterminant	0.78 (0.70, 0.86)	<0.001	0.76 (0.68, 0.86)	<0.001
Adequate	Reference	Reference	Reference	Reference
Adequate +	1.98 (1.87, 2.10)	<0.001	1.82 (1.70, 1.94)	<0.001
Pregnancy intention				
Intentional	Reference	Reference	Reference	Reference
Not intentional	0.82 (0.77, 0.87)	<0.001	0.91 (0.84, 0.99)	0.023
Unsure	0.92 (0.86, 1.00)	0.038	0.91 (0.83, 1.00)	0.044
Traditional smoker				
Yes	0.87 (0.08, 0.08)	<0.001	1.05 (0.92, 1.19)	0.486
No	Reference	Reference	Reference	Reference
Pre-pregnancy alcohol drink (number per week)				
None	Reference	Reference	Reference	Reference
<1	0.91 (0.85, 0.96)	0.002	0.94 (0.87, 1.01)	0.085
1-7	0.73 (0.69, 0.78)	<0.001	0.81 (0.75, 0.88)	<0.001
>7	0.80 (0.68, 0.95)	0.011	0.96 (0.80, 1.16)	0.693
Prenatal vitamin use (times per week)				
None	0.96 (0.90, 1.01)	0.100	1.06 (0.99, 1.14)	0.089
1-3 times	0.91 (0.82, 1.01)	0.074	0.97 (0.86, 1.09)	0.561
4-6 times	0.83 (0.74, 0.92)	<0.001	0.93 (0.82, 1.05)	0.226
Every day	Reference	Reference	Reference	Reference
Parity				
Primiparous	Reference	Reference	Reference	Reference
2nd child	1.12 (1.05, 1.19)	<0.001	0.88 (0.82, 0.95)	<0.001
3rd child	1.35 (1.25, 1.45)	<0.001	0.92 (0.84, 1.01)	0.066
4-6th child	1.57 (1.45, 1.70)	<0.001	0.90 (0.81, 1.01)	0.063
7th child +	1.60 (1.30, 1.97)	<0.001	0.86 (0.65, 1.12)	0.258
Preterm birth history				
Yes	0.59 (0.53, 0.65)	<0.001	1.33 (1.17, 1.52)	<0.001
No	Reference	Reference	Reference	Reference
Delivery method				
Vaginal	Reference	Reference	Reference	Reference
C-section	1.55 (1.47, 1.63)	<0.001	1.11 (1.05, 1.19)	<0.001
Year of delivery				
2016	Reference	Reference	Reference	Reference
2017	1.07 (0.97, 1.17)	0.185	1.02 (0.91, 1.13)	0.764
2018	1.03 (0.94, 1.12)	0.595	0.97 (0.87, 1.07)	0.506
2019	1.13 (1.03, 1.24)	0.008	1.06 (0.91, 1.18)	0.301
2020	1.27 (1.16, 1.38)	<0.001	1.172 (1.06, 1.30)	0.002
2021	1.26 (1.15, 1.38)	<0.001	1.17 (1.05, 1.30)	0.004

After adjusting for multiple confounders, secondary associations were discovered between maternal characteristics and GDM (Table 3). Namely, having an increased age at delivery, being Asian and Other races and Hispanic ethnicity, having an education level between 9th and 12th grade (versus high school graduate degree), being a WIC recipient, having a prenatal care Kotelchuck index of adequate plus, having preterm delivery history, higher BMI, delivery through C-section, and delivery in 2020 and 2021 (versus 2016) were associated with higher odds of GDM. 

## Discussion

Our findings contribute to the growing body of literature on EC use pre- and during pregnancy, specifically by identifying a higher adjusted odds of GDM in women who reported EC use pre-pregnancy only (OR: 1.27, 95% CI: 1.07-1.51, p = 0.006). Additionally, we found that EC use pre-/during pregnancy was uncommon among pregnant women with live singleton births from 2016 to 2021. Less than 5% of the 204,757 women in our study reported EC use pre- and/or during pregnancy, with the majority (72%) of those women discontinuing use before the third trimester.

Compared to non-users, EC users were more likely to be younger, unmarried, have lower educational attainment, experience unintentional pregnancies, not take prenatal vitamins, and have lower income levels. Consistent with prior research, we also found that pregnant women with GDM were more likely to be older, multiparous, Asian or Hispanic, have a higher BMI, and have lower education levels [14-16]. These demographic and clinical characteristics highlight potential areas for targeted prenatal counseling and intervention.

Recent studies have explored potential links between EC use and insulin resistance or diabetes mellitus (DM) [8,17,18]. Animal studies suggest that EC exposure may impair insulin tolerance by increasing TNF-α levels, which degrade insulin receptor substrates [17]. In human studies, findings have been mixed. A U.S.-based National Health and Nutrition Examination Survey (NHANES) study reported no direct association between EC use and DM but found increased insulin resistance among recent EC quitters (OR: 1.22, 95% CI: 1.00-1.64) [8]. Conversely, a study in a Korean population identified 15% increased odds of DM in EC users (OR: 1.15, 95% CI: 1.01-1.31) [18].

Findings from studies examining the relationship between traditional cigarette use and GDM have also been inconsistent [19-25]. While two meta-analyses found no significant association (OR: 1.06, 95% CI: 0.95-1.19; OR: 0.98, 95% CI: 0.88-1.10), other studies using U.S. and Scandinavian data reported increased odds of GDM (ORs ranging from 1.10 to 1.65) [19-23]. Interestingly, two studies found that women who quit EC use pre-pregnancy had higher odds of developing insulin-requiring GDM, though the retrospective nature of these studies introduces a potential reverse causality bias - where women may have been advised to quit smoking following a GDM diagnosis [24,25].

Our study similarly found that women who quit EC use before the third trimester had 27% higher odds of developing GDM compared to non-users (OR: 1.27, 95% CI: 1.07-1.51, p = 0.006), with no other significant associations observed across EC use categories. The mechanisms underlying this association remain unclear. One possibility is that nicotine cessation leads to short-term metabolic changes, including an increase in body weight and HbA1c within the first few years of cessation [26,27]. These metabolic changes could, in turn, increase the odds of developing GDM. Alternatively, reverse causality cannot be ruled out, as GDM is typically diagnosed between 24 and 28 weeks of gestation via an oral glucose tolerance test (OGTT) [28]. Since PRAMS only collects data on EC use in the three months pre-pregnancy and during the third trimester, it is unclear whether women quit EC use before or after their GDM diagnosis.

Clinical implications

These findings suggest that e-cigarette use before pregnancy may be a previously underrecognized risk factor for GDM. Given the increasing prevalence of e-cigarette use among women of reproductive age, clinicians should consider screening for e-cigarette use as part of preconception and early prenatal care. Counseling efforts should emphasize the potential metabolic risks associated with pre-pregnancy e-cigarette exposure, in addition to known fetal and neonatal concerns, to support informed decision-making and risk reduction in the prenatal setting.

Research implications

This study highlights the need for further prospective research to clarify the temporal relationship between e-cigarette use and GDM development. Future studies should aim to capture more granular data on the frequency, duration, and cessation timing of e-cigarette use, as well as nicotine concentration. Understanding these nuances will be critical to disentangling causality, informing mechanistic studies on nicotine’s metabolic effects during pregnancy, and guiding evidence-based policy and clinical guidelines regarding e-cigarette use in women of reproductive age.

Strengths and limitations

While this study benefits from the large, representative sample of the PRAMS database, several limitations in this retrospective secondary design must be acknowledged. First, the reliance on self-reported data introduces potential bias, particularly underreporting sensitive behaviors like smoking and e-cigarette use. Second, the data lacks details regarding EC use. The PRAMS questionnaire asked respondents about EC use three months pre-pregnancy and separately about EC use during the last three months of pregnancy. There was no EC use data for the first or second trimesters of pregnancy. Moreover, there were no questions regarding EC use frequency, nicotine concentration, or timing of use and/or cessation, therefore limiting the ability to evaluate the independent effects of e-cigarettes on pregnancy outcomes. Lastly, given that both EC use and GDM were relatively uncommon in our sample, statistical power to detect associations between continued EC use throughout pregnancy and GDM may have been limited.

## Conclusions

This study contributes to the growing body of literature on EC use during pregnancy by exploring its association with detrimental outcomes, specifically GDM. Our findings show evidence of an increased odds of GDM in women who used EC pre-pregnancy but quit before the third trimester. Given the limitations of retrospective analyses and self-reported data, further prospective research is needed to better understand the complex relationship between EC use and maternal metabolic health. Future studies should aim to collect detailed information on EC use patterns, nicotine concentration, and concurrent use of traditional cigarettes to clarify the independent effects of EC use on pregnancy outcomes. These efforts will be critical for informing public health policies and developing targeted interventions to mitigate risks associated with EC use during pregnancy.
